# Evolution and connectivity in the world-wide migration system of the mallard: Inferences from mitochondrial DNA

**DOI:** 10.1186/1471-2156-12-99

**Published:** 2011-11-17

**Authors:** Robert HS Kraus, Anne Zeddeman, Pim van Hooft, Dmitry Sartakov, Sergei A Soloviev, Ronald C Ydenberg, Herbert HT Prins

**Affiliations:** 1 Resource Ecology Group, Wageningen University, P.O. Box 47, 6700 AA, Wageningen, The Netherlands; 2 present address: Conservation Genetics Group, Senckenberg Research Institute and Natural History Museum, D-63571 Gelnhausen, Germany; 3 present address: Laboratory for Infectious Diseases and Screening (LIS) Centre for Infectious Disease Control, National Institute for Public Health and the Environment (RIVM), Bilthoven, The Netherlands; 4 Ecological Watch of Siberia, Komarova street 27/6/5, 644074 Omsk, Russia; 5 Department of Chemistry, Omsk State University, St. Prospect Mira 55a, 644077 Omsk, Russia; 6 Centre for Wildlife Ecology, Simon Fraser University, V5A 1S6 Burnaby BC, Canada

## Abstract

**Background:**

Main waterfowl migration systems are well understood through ringing activities. However, in mallards (*Anas platyrhynchos*) ringing studies suggest deviations from general migratory trends and traditions in waterfowl. Furthermore, surprisingly little is known about the population genetic structure of mallards, and studying it may yield insight into the spread of diseases such as Avian Influenza, and in management and conservation of wetlands. The study of evolution of genetic diversity and subsequent partitioning thereof during the last glaciation adds to ongoing discussions on the general evolution of waterfowl populations and flyway evolution. Hypothesised mallard flyways are tested explicitly by analysing mitochondrial mallard DNA from the whole northern hemisphere.

**Results:**

Phylogenetic analyses confirm two mitochondrial mallard clades. Genetic differentiation within Eurasia and North-America is low, on a continental scale, but large differences occur between these two land masses (*F*_ST _= 0.51). Half the genetic variance lies within sampling locations, and a negligible portion between currently recognised waterfowl flyways, within Eurasia and North-America. Analysis of molecular variance (AMOVA) at continent scale, incorporating sampling localities as smallest units, also shows the absence of population structure on the flyway level. Finally, demographic modelling by coalescence simulation proposes a split between Eurasia and North-America 43,000 to 74,000 years ago and strong population growth (~100fold) since then and little migration (not statistically different from zero).

**Conclusions:**

Based on this first complete assessment of the mallard's world-wide population genetic structure we confirm that no more than two mtDNA clades exist. Clade A is characteristic for Eurasia, and clade B for North-America although some representatives of clade A are also found in North-America. We explain this pattern by evaluating competing hypotheses and conclude that a complex mix of historical, recent and anthropogenic factors shaped the current mallard populations. We refute population classification based on flyways proposed by ornithologists and managers, because they seem to have little biological meaning. Our results have implications for wetland management and conservation, with special regard to the release of farmed mallards for hunting, as well as for the possible transmission of Avian Influenza by mallards due to migration.

## Background

The large-scale migration systems of temperate waterfowl (Anseriformes:Anatidae) have been extensively studied using ringing, telemetry, morphometrics, radar tracking and isotope analysis [1]. In general, migration routes are clearly defined [2]. Most have north-south bearings, with populations travelling between northerly breeding areas and more southerly non-breeding areas. Many species follow similar routes and decades of studies on bird migration have led to the delineation of major waterfowl 'flyways' [2-4]. Especially in North-America, flyways are managerial units created by agreements between adjoining states and provinces, and thus are bounded by management requirements. The boundaries of true migratory pathways (in a population ecological sense) are much fuzzier. Populations and individuals within species may occupy different flyways, and many migrants are flexible in migration routing [5], even though these migration routes have been in place for relatively long periods of time [6,7]. Especially in ducks, 'irregularities' in migration routes have been described, such as individuals switching migratory trajectories, termed 'abmigration' [8] or 'flyway permeability' [9].

The mallard (*Anas platyrhynchos*) is the most numerous Holarctic waterfowl species and distributed widely over the whole Northern Hemisphere. Northerly breeding birds are mostly migratory, wintering much further south, while birds breeding in temperate regions, especially in parts of Western Europe, can be resident [2]. Migratory mallard populations often do not exhibit clearly defined routes, even when breeding and non-breeding destinations are thousands of kilometres distant [10]. Mallards play a significant role in the management and conservation of wetland habitats [11], are a very common bird in recreational wetland parks as well as the major game species in wetland systems. Hunting of mallards is facilitated in many countries by supplementary restocking wild populations with farmed mallards [12], possibly with large-scale consequences for the population genetic structure, genetic integrity and fitness of the wild populations [13].

Due to its wide range and large population sizes, the mallard is considered the primary natural reservoir of avian influenza (AI) [14]. It has been identified (along with the black-headed gull *Larus ridibundus*) as the species posing the highest risk to transmit AI to farm-birds [15]. Mallards may (among other bird species) contribute to the spread of AI from Eurasia (Old World; OW) into North-America (New World; NW). Some studies propose trans-hemispheric movements of dabbling ducks between these land masses [16] and that these facilitate AI transmission [17,18] (but see [19,20]). The mallard is thus a prime candidate for spreading AI in the wild [21], and perhaps to humans, and it is important to learn more about its ecology, movements and dispersion [22,23]. Analyses of mitochondrial DNA (mtDNA) show just two genetic clades: clade A, mainly found in Asia, and clade B, found in North-America [10,24], indicating widespread mixing within but not between the Old and New World. However, to date no genetic studies have been carried out for the complete native range of mallard, as data from Europe and eastern North America were missing. A range-wide survey of genetic diversity and connectivity between proposed mallard flyways could thus be useful to finally generalise findings of small-scale population genetic and phylogeographic studies. Such a study would also add substance to an increasing body of literature on migration systems, flyways concepts, and genetic population structure in other waterfowl.

In this study, we attempt to close existing gaps in the knowledge of the genetic structure of female mallards by providing large numbers of mtDNA sequences from previously unsampled regions throughout the whole native distribution of the mallard. Due to the mallard's high mobility and examples of flyway permeability in closely related duck species, we hypothesise weak barriers to dispersal between Asia and Europe. Thus, in Europe we expect to find clade A haplotypes, as in Asia, and Europe and Asia to form a joint Eurasian 'Old World' meta-population, with possibly extensive east-west gene flow. Additionally, we present many more samples from central and eastern Canada, which lacked in the most recent analysis [10], to complete an mtDNA sequence data set comprising samples from the whole Holarctic. A combination of population genetic analyses and coalescent simulations in an isolation-with-migration framework [25,26] enables us in this to measure genetic diversity and geographic partitioning thereof, as well as population histories and migration rates between Old World and New World mallards, and to test the currently hypothesised flyways of mallards (see Table 1 and [2-4]).

**Table 1:** Sampling localities and genetic diversity

**land mass**^**1)**^	region	**flyway**^**2)**^	π (± SD)	dH (± SD)	locality	n	lat.	long.
OW	Europe	North-West EU	0.00344	0.696	GBAB	21	57.433	-2.393
			(0.00038)	(0.05)	NLFR	23	53.035	5.574
					NOBE*	22	60.355	5.345
		
		Central EU	0.00470	0.929	ATHO	18	48.607	16.905
			(0.00034)	(0.018)	DEWU	24	50.036	11.972
					EETA*	22	58.345	27.154
					RUKR	10	60.999	38.556
		
		East EU	0.00536	1.000	RUAS	3	46.217	47.767
			(0.00182)	(0.272)				
	
	Asia	Central Asia	0.00438	0.833	KZAO	3	44.893	75.122
			(0.00112)	(0.127)	RUOM*	6	55.845	71.853
		
		East Asia	0.00835	0.987	MNDA	1	47.000	119.367
			(0.00093)	(0.005)	RUKK*	4	50.388	136.996
					RUPR	1	59.631	149.115
					RUPR	82	45.007	132.432

NW	Alaska	Pacific NA	0.01323	0.985	USFI	13	64.825	-147.584
			(0.00151)	(0.015)	USIZ	6	55.358	-162.728
					USJU	1	58.364	-134.572
					USKB	2	60.545	-151.148
					USKI	4	57.491	-153.495
					USSF	1	61.216	-149.884
					USYD	1	61.367	-163.717
					USYR	1	65.821	-149.733
	
	Canada	Central NA	0.01364	0.971	CARM	20	50.628	-101.159
			(0.00175)	(0.021)	CASL*	3	49.666	-112.704
					USPI	1	72.677	-99.469
		
		Atlantic NA	0.01430	0.934	CACO	3	45.579	-64.345
			(0.00224)	(0.061)	CAJC*	2	42.321	-82.385
					CALM	9	43.962	-80.400

N/A	Aleutians	N/A	0.00870	0.824	USAD	2	51.762	-176.622
			(0.00243)	(0.084)	USAI	8	52.905	172.906
					USSI	7	52.723	174.112
	
	Greenland	N/A	0.00042	0.177	GLNU	22	64.190	-51.708
			(0.00027)	(0.106)				

NulceotieNucleotide diversity (π) and haplotype diversity (dH) were calculated per flyway. Sampling locations within each flyway are coded by the two letter country abbreviation (e.g., GB - Great Britain; NL - Netherlands, etc.) and a two letter locality abbreviation (GBAB - Great Britain, Aberdeen; NLFR - Netherlands, Friesland). Details on coding are given in Additional file 2. Samples sizes per locality within flyways (n) are reported, as well as digital GPS coordinates (latitude, lat. and longitude, long.; negative longitudes indicate westward direction).

^1)^land masses are defined as Old World (OW, which is Eurasia) and New World (NW, which is the North-American continent). The Aleutian Islands and Greenland are not assigned to either of these land masses

^2)^flyway definitions based on references 2-4

*coordinates are averages of several near-by places, where ducks have been sampled and combined into one sampling locality. Full details for each individual can be found in Additional file 2.

## Results

### MtDNA control region sequencing

Our data set is comprised of mtDNA sequences from 346 mallard ducks around the world consisting of 195 newly sampled mallards and 151 sequences from previous studies [10,27] (for details on the sequences and localities see Table 1 and methods section). 155 different haplotypes were found in this data set, of which 101 were already contained in the data set of Kulikova *et al*. [10,27], and 54 were novel. Of the 622 aligned nucleotide positions 93 (15%) were variable, 73 (11%) sites were parsimony informative and four sites showed gaps. Additional file 1 lists the haplotype names with corresponding sample IDs. Of the 155 haplotypes, 108 haplotypes were only represented by a single individual, 44 haplotypes by 2-7 individuals, one haplotype by 16 individuals (Hap 56), one haplotype by 20 individuals (Hap 57), and the most frequent haplotype (Hap A7) was represented by 71 individuals (see Additional file 1 for details). This A7 haplotype is found in both Old and New World, in almost all localities. All new sequences obtained in this study are deposited in GenBank [28] with accession numbers JN029963-JN030157. In Table 1 we further list nucleotide diversity (π) and haplotype diversity (dH). Nucleotide diversity is similar in all Old World flyways (π between 0.003 and 0.005, and 0.008 in Eastern Asia) but consistently higher in the New World (π > 0.013). Samples from the Aleutians have intermediate nucleotide diversity somewhere between Old and New World (π = 0.009). The Greenland samples are very low in both nucleotide diversity (π = 0.0004) and haplotype diversity (dH = 0.2), whereas haplotype diversity is high (dH > 0.8) in all other flyways except in the North-West Europe flyway (dH = 0.7).

Testing neutrality based on the frequency spectrum of the haplotypes with Fu's *F*_*S *_[29] revealed negative test statistics for both measures when samples were partitioned into Eurasia (*F*_*S *_= -25.52, p < 0.001) vs. North-America (*F*_*S *_= -24.63, p < 0.001) Splitting Eurasia into Europe (*F*_*S *_= -26.41, p < 0.001) and Asia (*F*_*S *_= -25.38, p < 0.001) leads to statistics significantly smaller than zero for both continents.

### Two distinct mallard clades

**Figure 1:**
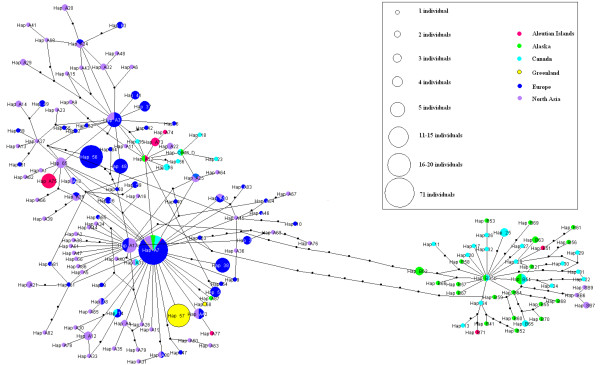
**Unrooted network illustrating the phylogenetic relationships of the mtDNA haplotypes**. Haplotype names correspond to the ones in Additional file 1 and colours to regions as indicated in Table 1. Circle sizes scale to indicate the number of individuals harbouring each haplotype. Small black dots indicate unsampled intermediate haplotypes. Note that distances between circles are not proportional to the genetic distance but are arranged for better visibility.

**Figure 2:**
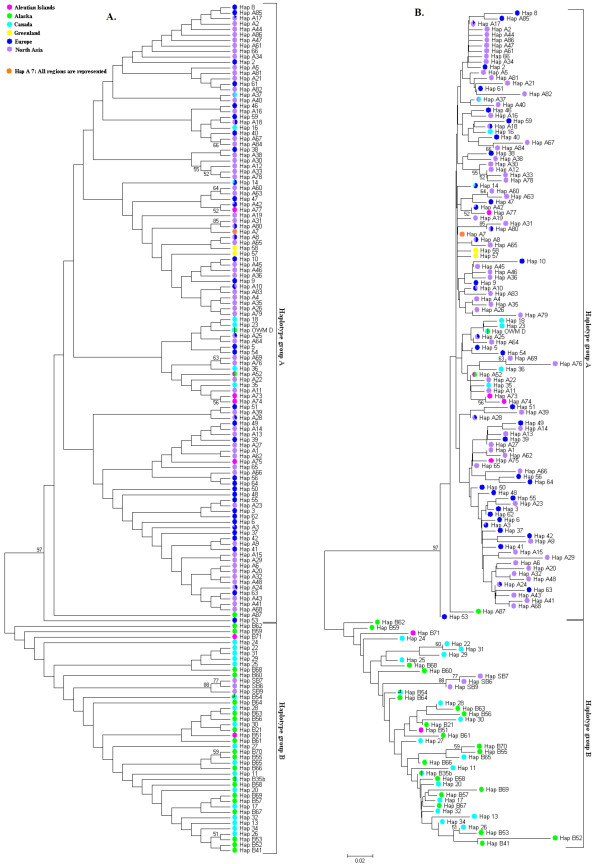
**Unrooted Neighbour Joining tree illustrating clade A and B mtDNA control region haplotypes**. Bootstrap values (500 replicates) are shown next to the branches if > 50%. Positions containing gaps are removed by pairwise deletion. Haplotype names and colours of the dots correspond to the ones in Figure 1. A: Tree ignoring branch lengths. B: Branch lengths scaled to evolutionary distances (sum of branch lengths = 2.65).

An unrooted haplotype network (Figure 1) shows the presence of two distinct clades. These clades correspond well to a classification of sampling locations into Old World (clade A haplotypes, mainly Eurasia) and New World (clade B haplotypes, mainly North-America). Within these clades, a great variety of haplotypes was sampled with relatively few missing intermediate ones, represented by small black dots in Figure 1. Clade A and clade B are separated by about ten substitutions. Phylogenetic inference by building a Neighbour-Joining [30] tree supported the split between clade A and clade B haplotypes with 97% of the bootstrap replicates (Figure 2).

### Low genetic differentiation within Eurasia and North-America, but large differences between them

Genetic differentiation between Eurasia and North-America (Aleutians and Greenland excluded, see methods section) and between all individual flyways (flyways definitions are in Table 1) was assessed by Wright's *F*-statistics [31]. The *F*_ST _value between the two land masses was 0.51. Pairwise *F*_ST _values on the flyway-level are given in Table 2. Not all flyways were significantly differentiated from each other. In case of the Eastern Europe flyway sample size was too low (n = 3) to make any meaningful statement about the amount of differentiation. None of the intra-North-American flyways was significantly differentiated from the others on that continent. The same was found between the two Asian flyways. In Europe, however, with the exception of the Eastern European flyway which suffered from low sample size, we observed significant structure, albeit low in magnitude (North-West vs. Central Europe: *F*_ST _= 0.08). Notably, the Aleutian sample was most differentiated from the North-American Pacific and Central flyways but to a lesser extent from the Eurasian ones (in some cases not even significantly; Table 2). Finally, the Greenland sampling location displayed relatively high and significant differentiation with each of the other flyways.

**Table 2:** Pairwise *F*_ST _values for all flyways

	1	2	3	4	5	6	7	8	9	10
1) Pacific NA	-									
2) Central NA	-0.01	-								
3) Atlantic NA	0.19	0.15	-							
4) North-West EU	0.65*	0.63*	0.33*	-						
5) Central EU	0.62*	0.60*	0.28*	0.08*	-					
6) East EU^1)^	0.44	0.39	0.03	0.05	-0.09	-				
7) Central Asia	0.50*	0.47*	0.16	0.08	0.00	-0.07	-			
8) East Asia	0.52*	0.49*	0.16*	0.06*	0.01	-0.12	-0.01	-		
9) Greenland	0.64*	0.64*	0.41*	0.36*	0.30*	0.71*	0.54*	0.18	-	
10) Aleutians	0.43*	0.40*	0.10	0.17*	0.09*	-0.06	0.05	0.05	0.37*	-

*F*_ST _values printed boldface and marked by an asterisk are statistically significant after Bonferroni correction.

^1)^note, that the east European flyway is only represented by three samples, and hence the presented values are very crude!

An analysis of molecular variance (AMOVA) revealed little genetic variation within the various Eurasian or North-American flyways compared to the amount of variation between these two land masses. 50.2% of the genetic variation lies between North-America and Eurasia, and 46.6% within the sampled localities. Only 3.2% of the genetic variation was partitioned between flyways within the continents (Table 3). Performing an AMOVA in which localities are not pooled into flyways does not lead to qualitative differences of this outcome (Table 3; values in brackets).

**Table 3:** AMOVA analysis of flyway genetic variance in the land masses Eurasia and North America

	d.f.		SS		VC		% var	
Between land masses	1	(1)	288.03	(288.03)	2.67	(2.66)	50.20	(49.94)
Between flyways (localities) within land masses	6	(26)	50.71	(168.71)	0.17	(0.43)	3.22	(8.1)
Within flyways	299	(279)	740.26	(622.74)	2.48	(2.23)	46.57	(41.96)
Total	306	1079		5.32	100			

Values in brackets were obtained when treating sampling localities directly as groups (i.e., not pooled into flyways). All variances are statistically significant (p < 0.05). Abbreviations: d.f.: degrees of freedom, SS: sum of squares, VC: variance components, % var: percentage of variation.

When we assigned localities to flyways within continents, per-continent AMOVA showed that genetic variation was to a large extent partitioned within localities: 85% in North-America, 76.8% in Europe, 98.7% in Asia and if combined into one land mass: 87.7% in Eurasia. In none of these analyses there was a significant amount of genetic variation between flyways (p > 0.2). In Europe, there was a significant (p < 0.001) genetic variance component of 22.9% between the localities within the flyways, in the other continents this was not statistically significant.

### Demographic history and gene flow

The runs of the coalescent simulation program IMa2 converged quickly in an optimal parameter space. Effective sample sizes for all parameters exceeded 300,000 and each newly sampled genealogy was independent from the previous one (autocorrelations in all parameters were 0.05 and less). Pairwise correlations of parameters were between -0.1 and +0.1, except between q0 and q1 (the estimates of population size for Old World (OW) and New World (NW)) where it was -0.19.

**Figure 3:**
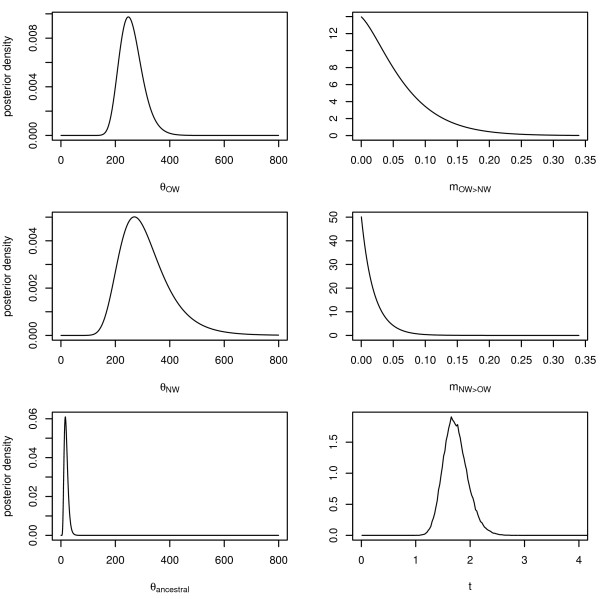
**Density plots of the posterior probability distribution for each of the six estimated demographic parameters**. Separate estimates for the effective population size (theta; θ) are given for Old World (OW), New World (NW) and the ancestral population, as well as the splitting time (t). Migration rates from OW into NW (abbreviated as 'm_OW→NW_') and the opposite direction are also given. Note that all estimates are scaled to mutation rate and not in demographic units; migration rates are displayed in the direction of individual movements (i.e., not in the coalescent notation as presented in the raw output of the IMa2 program).

All parameter estimates had relatively sharp unimodal posterior density distributions (Figure 3), except the migration rates which were poorly estimated, with wide ranges covering three orders of magnitude (m_OW→NW _and m_OW→NW_: 5.1 × 10^-9 ^- 4.7 × 10^-6 ^and 5.1 × 10^-9 ^- 1.8 × 10^-6^, see Figure 3). Table 4 summarises the results in demographic units. Although 95% highest posterior densities (HPD95) for population sizes of OW and NW samples overlapped IMa2 calculated that there was a 66% probability that the effective population size of the NW mallards (1.3 - 4.2 million) is larger than the OW population (1.5 - 2.9 million). Further, even though the HPD95 ranges of the migration rates peaked almost at zero, there was a 76% probability that the migration rate from OW into NW is higher than the other way around in the few cases in which it may occur.

**Table 4:** Demographic parameters as estimated under an isolation with migration model

parameter	scaled to mutation rate			in demographic units		
	**mode**	**HPD95L**	**HPD95H**	**mode**	**HPD95L**	**HPD95H**

θ_ow_	247.6	181.2	345.2	2,073,285	1,517,283	2,890,541
θ_NW_	270.0	154.8	499.6	2,260,852	1,296,222	4,183,414
θ_ancestral_	16.4	6.8	35.6	137,326	56,940	298,098
t	1.7	1.3	2.2	55,366	42,002	74,993
m_OW→NW_	0.00017	0	0.1586	5.1 × 10^-^^9^	0	4.7 × 10^-^^6^
m_NW→OW_	0.00017	0	0.06069	5.1 × 10^-^^9^	0	1.8 × 10^-^^6^

For calculation of demographic units a generation time of one year was used. The mutation rate per locus per year was set to 2.9856 × 10-5. See main text for further explanations. The demographic units are: population size (θ in effective individuals, splitting time (t) in years, migration rates (Nm) in effective individuals per year. Estimates of the peak of the posterior parameter distributions (mode) are presented along with low and high bounds of the 95% highest posterior densities (HPD95L/H).

## Discussion

### Conclusive assertion: There are two distinct mallard clades

Since Avise *et al*. first studied genetic structure in mallards in 1990 [24] it is hypothesised that no more than two mitochondrially distinct clades exist among them. Our phylogenetic analyses confirm this hypothesis for mallard samples collected and analysed from the numerically largest and geographically most complete set of mallard mtDNA sequences to date. In addition to previously analysed North-American and Asian samples [10], we added substantial numbers of European and Central and Eastern North-American mallard samples and thus completed a data set spanning the whole Northern Hemisphere. Through large-scale sampling of mallards from all over their distribution range we can now conclusively state that no more than two main clades exist in mallard, also not in the previously unsampled European populations.

### Gene pool differentiation and diversity

Mallards are highly mobile and some studies suggest that natal philopatry (i.e., philopatry in the sense of [32]), which in waterfowl is usually strongest in females, is less pronounced - even in female mallards - than it is in related species [2,9,33] (but see [34-36]). We found strong support for the hypothesis of 'flyway permeability' [8,9] in mallards. The analyses of *F*_ST _between intra-continental flyways strongly promote the conclusion of female inter-flyway mixing. Neither within North-America nor in Asia did we detect differentiation between the subcontinental flyways. We note that for some comparisons the sample size was on the low side. However, if this was causal to not finding significant differentiation between flyways within a continent, we would also not have found significant differentiation between those flyways with lower sample sizes with flyways on different continents. Hence, we believe our finding of a lack of differentiation is valid. Yet, there is population structure in Europe based on *F*_ST _analysis (see below).

Confirmative evidence for the pattern of genetic population structuring found above stems from our analysis of molecular variance (AMOVA). Almost no genetic variation in our world-wide data set is explained between flyways within continents. On this global scale, about half the variation lies between North-America and Eurasia, and the other half within the flyways. When analysing genetic variance per continent, in North-America and Asia the largest proportion of variance resides on the level of sampling localities but not within or between flyways. However, notably, in Europe there is an unexpected significant variance component between localities within flyways, pointing at more genetic structure than in the other continents. In general, these findings confirm the absence of strong female philopatry in mallards. The population structure is not shaped by female mallards returning to the place where they were born, and hence lifetime dispersal seems high. In that sense, currently recognised waterfowl flyways do not depict well the movements and dispersal of mallards - a pattern similar to other duck species [33,37,38] - although it cannot be generalized for all ducks [39,40]. However, our findings show that indeed the European mallard population seems to be more structured than their conspecifics in other parts of the world. Geographic, micro-climatic and urbanisation structuring in Europe may be different in Europe when compared to Asia and North-America, shaping mallard population structures in different ways. Future studies employing genetic markers, probably at an even denser sampling scheme, may be able to quantify such effects by making use of GIS and remote sensing technology. A further possibility to explain European mallard population structure may be extensive release of farmed mallards for hunting purposes in many countries in Europe. These originally wild mallards are bred in captivity for many generations, and even though looking quite similar to their wild relatives they can differ subtly in morphology [12]. Such differences have been proven to leak into wild populations by interbreeding with escaped farmed mallards [41]. Obviously, this introgression of genetic material of farmed animals has consequences for the genetic identity of wild populations, especially when translocation and release at distant localities takes place [42]. Genetic introgression has been demonstrated for mallards in Italy already [43]. Such manipulation of local population structure may well explain our finding of increased genetic structure in Europe.

### Demographic history

Mallards from the Old World are different from those in the New World, which is attributable to the differences in the distribution of clade A (Eurasia) and clade B (North-America) haplotypes. In Europe and the largest part of Asia we only found clade A haplotypes with the exception of a handful clade B haplotypes in the far east of Asia (Figures 1, 2). Nevertheless, in a phylogenetic assessment in the original study in which these sequences were determined, they were shown to be characteristic for introgressed eastern spot-billed ducks and likely do not indicate original North-American mallard clade B individuals [10,27] (removing these three curious sequences from the demographic modelling analysis did not alter the results, though; data not shown). On the other hand, in North-America, where clade B dominates, also clade A haplotypes are relatively common, which is reflected in higher nucleotide and haplotype diversities. This suggests two factors shaping the current genetic structure in mallards. From the deep split into clade A and B haplotypes we infer that Old World and New World have become separate with no or little gene flow since then. Further, if genetic exchange occurs - as indicated by clade A haplotypes occurring both in Old and New World - it seems directional with larger rates from Old World into New World. However, this directionality is not statistically robust because in the IM analysis migration rates were only estimated poorly. The split between mallards from Old World and New World was estimated to have occurred between 43,000 and 74,000 years ago. This dating coincides with the drop in sea level during the last glacial period about 110,000 - 10,000 years ago. During this time the Beringia land bridge fell dry and extensive exchange of floras and faunas became possible. When the glaciers melted again, this Beringia land bridge connection got cut off and Eurasian and North-American faunas and floras became separated more strictly. But also during the glacial maximum the continental ice sheet developed in North-America separated northern from southern regions of this continent [44]. The estimated population size at that time (θ_ancestral_) was about 30 times smaller than it is today (the sum of θ_OW _and θ_NW_). Such high growth rates in waterfowl populations since the last glaciation has already been demonstrated in several other studies, often proven to be exponential [10,16,33,45] and connected to population size bottlenecks. This could explain the excess of rare haplotypes as demonstrated especially by negative Fu's *F*_*S *_in Eurasia, but also in North-America. The present-day situation is a deep split into an Old World and New World population, with migration rate estimates statistically not significantly different from zero, indicating that the rise of sea levels after the last glacial maximum results in a potent barrier to female gene flow between OW and NW. Numerically, the peak of the density distribution of migration rates suggests 0.04 - 0.05 effective migrants per year in each direction. However, the posterior density plots of the migration rates (Figure 3) are not bell-shaped as they should be. This indicates that IMa2 did not find enough information in the data to reliably estimate migration rates. If prior ranges are too wide, software like IMa2 may estimate parameters to be zero, but in our analysis the range of the prior distribution (0 - 0.34) was not much wider than the range of the estimated posterior distribution (HPD95) for migration of Old World mallards to the New World (0 - 0.16) (Table 4). Re-running the IMa2 analysis with a narrower prior (0 - 0.2) did not resolve the issue (data not shown). We believe this is due to a combination of very large extant population sizes as a result of strong population growth and divergence. Based on HPD95 migration rates could be as low as zero, or as high as 23 (NW→OW) or 80 (OW→NW) effective individuals. The failure of IMa2 to quantify migration rates may be related to this issue. Irrespective of this, the sharing of identical haplotypes indicates the possibility that migrants do travel between the land masses and establish genetic traces in the receiving population. The nucleotide diversity of the Aleutian samples, being intermediate between that of Eurasia and North-America, is a good indicator for this. Even though migration rates as such were poorly estimated with our data, the shapes of the migration rate curves and the implemented probability test in IMa2 point at directionality of migration from Old World into New World.

### Genetic exchange between Old World and New World

Based on the sharing of haplotypes between Old and New World mallards and the results of the demographic modelling, we propose that some genetic exchange of mtDNA between the two land masses possibly occurs. IMa2's estimated migration rates, being not statistically different from zero, may be biased downwards due to a lack of statistical power for the migration rate parameters, or model violation of migration/drift equilibrium, but indicate directionality. Genetic differentiation between Old and New World was high (*F*_ST _= 0.51) indicating limited gene flow at most. We propose that the route of this migration is mainly, if not completely, via the Aleutian Islands. On these islands individuals with clade A and B haplotypes intermix and form a haplotype cline [10], and genetic diversity (π) is intermediate between Eurasia and North-America. *F*_ST _values between the Aleutians and the flyways indicate a closer relationship between the Aleutians and Old World populations than with the New World. This may be explained by the Westerly winds (more regularly blowing from the west into the east), governing the Ferrel cell of the global climatic circulation system. The Aleutian Islands are all well within the Ferrel cell that reaches up to higher than 60° north [46,47], and characteristic winds are especially strong during the time in which mallards migrate [48]. A note of caution is warranted here, because smaller scale terrestrial weather conditions can be more important than the large atmospheric systems. Even if weak when compared to the strength of flight abilities this effect can have a profound impact on the regular "drifting off" of migrating birds in general [48] and waterfowl specifically [49].

The alternative route, from Europe via the Atlantic Ocean, along Iceland and Greenland, could be deemed less likely on basis of this "drifting by wind" proposition. Mallards travelling to North-America by the Atlantic route would face headwinds more regularly [48]. However, the mallards sampled from Greenland all bear clade A haplotypes implying a Eurasian origin. Additionally, genetic differentiation between mallards from the Atlantic part of Canada and European mallards is only half of what we measured between central or western Canada and Europe. Once arrived in Greenland it would not be hard to imagine crossing the last few hundred kilometres to Canada. Unfortunately, we were not able to analyse more samples from Greenland, especially from different localities. Interpretations based on the current Greenland data can only be tentative. Just two different haplotypes were found there and the sampling location was a pond near the city of Nuuk, likely inhabited by closely related mallard families. Little is known about the origin and movements of Greenland mallards [2,50], and hardly anything (except from data in this study) about their genetics (for a preliminary report, see [51]). More studies into this population are certainly needed to draw firm conclusions. Sampling of mallards from Iceland and the Faroe Islands would make it possible to perform detailed analyses of a potential Atlantic route of genetic exchange of mallard populations. Note, however, that all results presented in this paper are based on the analysis of mtDNA which is a maternally inherited marker. Patterns discerned from our data are thus only valid for the female part of the population. Males are sometimes suggested to also possess a homing instinct [52] but in contrast to females this is generally believed to be much less pronounced [53]. It will thus be important to study nuclear markers in mallard [43,54-57] on large geographical scales which depict the genetic structure of both sexes (Kraus *et al.*, manuscript in preparation). Some sex-bias in migration is not unusual [58] but can be extreme if males are much more dispersive than females, as demonstrated in a famous example of the white shark *Carcharodon carcharias *[59].

### Post-glacial colonization

Clade A is characteristic for Eurasia, and clade B for North-America, although many individuals in North-America belong to clade A. Kulikova *et al*. [10] proposed two phylogeographic hypotheses to explain such a haplotype distribution, termed "Asian invasion" and "incomplete lineage sorting" (initially suggested by Avise *et al*. [24]). In an Asian invasion scenario the occurrence of clade B haplotypes in mallards is explained by Asian mallards moving into a previously mallard-free North-America, followed by acquiring B-haplotypes by introgressive hybridisation with closely related indigenous duck species (such as the black duck *A. rubripes *[60]) resulting in frequently observed mtDNA paraphyly [27,61,62]. The incomplete lineage sorting hypothesis rests on the proposed occurrence of a polymorphic ancestral gene pool (at least with respect to mtDNA clades) which is facilitated further by large populations [63,64] and [Kraus *et al*., manuscript submitted]. These two hypotheses offer different predictions about the expected distribution of clade A and clade B haplotypes in North-America: As a result of an Asian invasion one would expect to find a gradual decline of clade A haplotypes from western to eastern North-America if we assume that all immigration from Eurasia took place via the Aleutian Islands and continues so. We cannot confirm this scenario; clade A haplotypes today occur in the whole of North-America. This, however, can also be the result of large dispersal abilities of mallards in North-America, which can diminish genetic clines relative quickly. Hence, an Asian invasion cannot be clearly rejected, either. Kulikova *et al*. [10] imagine this further to be due the impact of mallard farming, resulting in anthropogenic translocation also of clade A mallards. This explanation resembles one of the possibilities we propose to explain genetic structure in Europe.

As an alteration of the Asian invasion scenario we offer additional thoughts based on the extent of the most recent continental glaciation in North-America. The opening of the Beringia land bridge on the one hand enabled exchange of fauna and flora between East Asia and Alaska because these regions were to a large extent ice-free. Coincident with the emergence of this connection, though, the considerable North-America ice sheet built up south of Alaska, what is today southern Canada. An Asian invasion thus took place probably only in the very north of North-America. The clade B haplotype may have become fixed south of that ice sheet due to the population bottlenecks. After the retreat of the glaciers clade B mallards from the south got into secondary contact with northern clade A mallards. It would thus be interesting to sample mallards from central and southern North-America with this hypothesis in mind. To account for and further study mtDNA paraphyly in duck species such a study would have to include samples especially from Black Ducks [24,60], and if possible from other *Anas *species harbouring clade B haplotypes, too [61,62]. Avise *et al.*'s incomplete lineage sorting hypothesis [24] would naturally account for a more even distribution of clades and is consistent with the idea that a dichromatic (and mitochondrially polymorphic) mallard population gave rise to its monochromatic sister species by peripatric isolation [65]. But as Kulikova *et al*. [10] point out it seems unlikely that only the clade B haplotype gets fixed in all sister species but not the "original" mallard. We thus concur with Kulikova *et al*. [10] in concluding that a complicated mix of historical, recent and anthropogenic factors shaped the current world-wide mallard population structure. The results we can add from analysing >100 additional European mallards and the outcome of our demographic modelling study substantiate this claim.

## Conclusions

Many aspects of the biology of ducks are known when it concerns ecological and management parameters (see [11] and references therein for a recent overview), and also their phylogenetic placement has frequently been assessed, e.g. [61,66]. Surprisingly, only few studies have contributed to discern population genetic patterns for ducks, especially mallards. The information collected in this study is essentially representing the first complete assessment of the world-wide mallard population genetic structure in this duck. With more than 300 samples from the whole distribution range of the mallard we do not find intermediate haplotypes between clade A and B, or additional discernable clades. Our data and analyses corroborate the conclusion of Kulikova *et al*. [10] that a complicated mix of historical, recent and anthropogenic factors shaped the current world-wide mallard population structure. Further, we offer an additional hypothesis on how the current haplotype distribution emerged: a partial Asian invasion that took place only in northern North-America, which was ice-free at the last glacial maximum, followed by secondary contact with the southerly mallard population in North-America in which clade B haplotypes could have been fixed. A study to address this hypothesis would need to additionally analyse mallard samples from several well-spaced localities throughput central and southern North-America, as well to pay attention to *Anas *species closely related to mallards, often bearing clade B haplotypes themselves [61,62]. Mallards form an enormously large population. In the northern hemisphere, the population is structured deeply into two major clades, but within these landmasses the populations are very homogenous (and perhaps panmictic) in which the concept of flyway do not contribute to a further understanding of mallard population genetic structure. Homogeneity over thousands of kilometres facilitates the spread of diseases such as Avian Influenza. Between the two landmasses there may be a little gene flow, apparently in west - east direction across the Bering Strait. The genetic distinction between the OW and the NW is not eroded away by this gene flow, and apparently arose during the last Glacial Maximum.

Even though we refute the flyway concept for mallards as a representation of biological realities of geographic structuring of populations, we would like to point out that waterfowl can very well be managed on the basis of a flyway concept: the North American success story of waterfowl population increases over the last century underpins that success. In that sense, flyways ought to be viewed as 'problemsheds' in the sense of the Ecosystem Approach of the Convention on Biological Diversity and not so much as biological realities.

## Methods

### Sample collection

Blood from 195 mallards was collected on FTA cards [67], in most cases by hunters. Exceptions are the localities from Greenland and Norway. There, mallards were trapped, blood drops on FTA cards were sampled from the wing or foot vein, and mallards were released again (animal handling approved by the animal ethical committee of Wageningen University - DEC, the Greenland Home Rule and the Norwegian Food Safety Authority - Forsöksdyrutvalget). Details on sampling localities and samples can be found in Table 1 and Additional file 2. A cautionary note is needed for the Greenland samples. The trapping of these animals most likely resulted in the catching of a few ducks with several of their chicks which often entered the trap as a group. This may have affected some measures of population genetic parameter (see results and discussion).

For phylogenetic analyses samples were assigned to continents to visualise their geographic region of origin. Greenland and the Aleutian Islands were not assigned to a specific continent, and Alaska and Canada were treated as separate regions within North-America. For population genetic analyses, sampling localities were pooled into biological populations based on hypothesised mallard flyways in Europe [2], Asia [3], and North-America [4] (Table 1), allowing us to test our data against these human-made classifications. Samples from Greenland and the Aleutian Islands were classified as separate populations for their intermediate status.

### DNA isolation and sequencing

DNA was extracted from FTA cards using the Gentra Systems 'Puregene DNA purification Kit' (Qiagen, Valencia, California). The manufacturer's instructions were followed with slight modifications for handling the FTA cards: up to a quarter of the encircled area of the FTA cards (depending on how much blood was preserved) was cut into small pieces of approximately 2 mm^2 ^and digested with 60 μg Proteinase K (Sigma- Aldrich, St. Louis, Missouri) in 600 μl Cell Lysis Solution (Gentra Systems) at 55°C over night. Subsequently, proteins were precipitated with 200 μl Protein Precipitation Solution (Gentra Systems) and spun down together with the FTA card material. DNA in the supernatant was precipitated with isopropanol and washed with 70% ethanol. Quantity and purity of the DNA were measured using a Nanodrop ND1000.

PCR amplification targeted the 5'end of the mtDNA control region which is homologous to positions 79-773 in the chicken (*Gallus gallus*) mitochondrial genome [68]. In some duck species the presence of 'numts' (nuclear copies of mtDNA) was proposed in this region [69] but previous examination of mallard sequences did not reveal evidence for this [10]. Reactions were performed in 12 μl containing 30 ng genomic DNA as template, 3 μl STE buffer, 5.5 μl Abgene Mastermix (ThermoScientific) and 0.25 μl of each primer (10 mM): L78 [69] (forward) and H774 [70] (reverse). PCR amplification was done in a BioMetra Thermocycler (Biometra, Göttingen, Germany) under the following cycling conditions: 7 minutes initial denaturation at 94°C, followed by 45 cycles of 20 seconds at 94°C, 20 seconds at 49°C and 1 minute at 72°C, completed by 7 minutes final elongation at 72°C. Quality and quantity of the PCR product was determined by gel electrophoresis and the product was purified by vacuum filtration on a Millipore Multiscreen PCR plate. Forward and reverse DNA strands were cycle-sequenced using the ABI Big Dye Terminator Cycle Sequencing Kit 3.1 under the following cycling conditions: 1 minute initial denaturation at 96°C followed by 25 cycles of 10 seconds at 96°C, 5 seconds at 49°C, and final elongation of 4 minutes at 60°C. The sequencing reaction products were precipitated by sodium acetate and ethanol to purify the product, followed by capillary sequencing on an ABI 3730 DNA Analyzer. The forward sequences were verified with the sequence of the reverse strand in MEGA4 [71]; some manual corrections where needed. If the forward sequence was absent, or only partially resolved, the reverse strand was used and aligned with the other sequences for verification. Additionally, 151 published sequences from studies of Kulikova *et al*. [10,27] were downloaded from GenBank [28] (accession numbers: AY506868-AY506870; AY506873-AY506901; AY506904-AY506908; AY506910-AY506917; AY506919-AY506944; AY506974-AY506984; AY928831-AY928899). Altogether, these sequences were aligned in MEGA4 [71] using the ClustalW algorithm [72] under default settings. Names of haplotypes defined in the studies by Kulikova *et al*. [10,27] were preserved.

### Phylogenetic analyses

A phylogenetic tree of the haplotype sequences was constructed in MEGA4 [71], using the Neighbour-Joining algorithm [30] with 500 bootstrap replicates. Evolutionary distances were computed under the Tajima Nei model [73]. All positions containing alignment gaps and missing data were eliminated from the dataset for tree construction (complete deletion option). Further, a phylogenetic network was constructed in TCS [74] (version 1.21) by statistical parsimony, here, treating alignment gaps as fifth state.

### Population genetic analyses

The basic population genetic parameters nucleotide diversity (π) and haplotype diversity (dH) for each flyway was calculated with DnaSP [75]. Fu's *F*_*S *_[29] was calculated in Arlequin 3.5.1.2 [76], and evaluated for statistical significance by 16,000 simulated samples in order to guarantee a less than 1% difference with the exact probability in 99% of the cases [77]. Population differentiation was assessed by Wright's *F*-statistics [31], and partitioning of genetic variance among and within groups was investigated by analyses of molecular variance (AMOVA [78-80]). Calculations were also performed in Arlequin 3.5.1.2 [76] from pairwise nucleotide differences, and statistical significance tested by 16,000 permutations.

### Demographic modelling

To make inferences about the extent of migration between Old World (OW) and New World (NW) we modelled the demographic history of mallards by coalescent simulations under an "Isolation with Migration" (IM) model [25,26], as implemented in the program IMa2 (Linux version 10.13.10). OW and NW samples were treated as belonging to distinct populations based on their sampling locality. Greenland and Aleutian samples were excluded because of their possible intermediate status. Upper bounds for parameter priors were estimated during consecutive preliminary runs of the program, based on initial estimates of theta as advised in the manual of IMa2. The final values used for population size, migration rate and splitting time were: -q 800, -m 0.34, -t 23.448. We ran 60 Markov chains in parallel under a geometric heating scheme (option -hfg), with the hottest chain being *β *= 0.5 and the coldest chain *β *= 0.975. Estimated parameters in IMa2 are scaled to the mutation rate. To convert them into demographic units we used a mutation rate of 4.8 × 10^-8 ^(confidence interval 3.1-6.9 × 10^-8^) substitutions per site per year initially published for a wood duck [39]. This rate also produced sensible results in a study of two other ducks of the genus *Anas *[81,82] and needs to be multiplied by the number of nucleotides in the sequence alignment (here, 622) to obtain the substitutions per locus per year to be used for IM analysis. From the two sequence mutation models available in IMa2 we chose HKY [83] which is the applicable model for mtDNA control region sequences [84]. Several run time settings with different heating schemes and durations were explored, all yielding essentially the same outcome. The final simulation, for which we report the results here, was run for a burn-in period of 360,000 steps (they reached convergence already after a few 10,000 steps), and afterwards 26,000 genealogies were sampled every 100 steps from a total 2,600,000 steps.

## Authors' contributions

RHSK and AZ designed the study, carried out and coordinated sampling efforts, analysed the data and wrote the paper. PvH designed the study, analysed the data and wrote the paper. DS and SAS coordinated sample collection and discussed the paper. HHTP and RCY revised the manuscript. All authors read and approved this paper.

## Supplementary Material

Additional file 1 **MS Excel sheet giving information which samples had which haplotype**. Click here for file

Additional file 2 **MS Excel sheet with all details for each mallard samples and sequenced for this study**. Details given include internal sampling ID, sampling date, sampling country, names of sample collectors, name of sampling location, decimal latitude and longitude coordinates and determined sex of the sampled mallard. Click here for file
